# Combined inactivation of the SOS response with TCA fumarases and the adaptive response enhances antibiotic susceptibility against *Escherichia coli*

**DOI:** 10.3389/fmicb.2025.1570764

**Published:** 2025-05-09

**Authors:** Marina Murillo-Torres, Isabel María Peñalver-Fernández, Marta Quero-Delgado, Sara Diaz-Diaz, María Romero-Muñoz, Esther Recacha, Fernando Docobo-Pérez, José Manuel Rodríguez-Martínez

**Affiliations:** ^1^Departamento de Microbiología, Facultad de Medicina, Universidad de Sevilla, Sevilla, Spain; ^2^Instituto de Biomedicina de Sevilla (IBIS), Hospital Universitario Virgen Macarena / CSIC / Universidad de Sevilla, Sevilla, Spain; ^3^Unidad Clínica de Enfermedades Infecciosas y Microbiología, Hospital Universitario Virgen Macarena, Sevilla, Spain; ^4^Centro de Investigación Biomédica en Red en Enfermedades Infecciosas (CIBERINFEC), Instituto de Salud Carlos III, Madrid, Spain

**Keywords:** antibiotic stress, resistance reversion, DNA damage response, fumarases, adaptive response, SOS response

## Abstract

**Introduction:**

Targeting bacterial DNA damage responses such as the SOS response represents a promising strategy for enhancing the efficacy of existing antimicrobials. This study focuses on a recently discovered DNA damage response mechanism involving tricarboxylic acid cycle (TCA) fumarases and the adaptive response, crucial for *Escherichia coli* survival in the presence of genotoxic methyl methanesulfonate (MMS). We investigated whether this pathway contributes to protection against antibiotics, either separately or in combination with the SOS response.

**Methods:**

An isogenic collection of *E. coli* BW25113 mutants was used, including strains deficient in fumarases (Δ*fumA*, Δ*fumB*, Δ*fumC*) and the adaptive response (Δ*alkA*, Δ*alkB,* Δ*aidB*). Additional SOS response inactivation (Δ*recA*) was conducted by P1 phage transduction. All mutants were subjected to antimicrobial susceptibility testing, growth curve analysis, survival and evolution assays. To validate the relevance of these findings, experiments were also performed in a quinolone-resistant *E. coli* ST131 clinical isolate.

**Results and discussion:**

Overall, no significant differences or only moderate increases in susceptibility were observed in the single mutants, with Δ*fumC* and Δ*aidB* mutants showing the highest susceptibility. To enhance this effect, these genes were then inactivated in combination with the SOS response by constructing Δ*fumC*/Δ*recA* and Δ*aidB*/Δ*recA* mutants. These combinations exhibited significant differences in susceptibility to various antimicrobials, particularly cephalosporins and quinolones, and especially in the Δ*fumC*/Δ*recA* strain. To further assess these results, we constructed an *E. coli* ST131 Δ*fumC*/Δ*recA* mutant, in which a similar trend was observed. Together, these findings suggest that co-targeting the SOS response together with fumarases or the adaptive response could enhance the effectiveness of antibiotics against *E. coli*, potentially leading to new therapeutic strategies.

## 1 Introduction

Antibiotic resistance has emerged as a significant challenge to human health in recent decades. The widespread and often inappropriate use of antibiotics has accelerated the continous evolution of diverse resistance mechanisms, enabling bacteria to evade the effects of these drugs (Cook and Wright, 2022). A better understanding of the molecular responses triggered in bacteria under antibiotic pressure is essential to address this situation. Such insights would help to identify new bacterial targets for the development of novel antimicrobial compounds, as well as the optimisation of existing antibiotic therapies (Baker et al., 2018; Blázquez et al., 2018; Stokes et al., 2019).

Antibiotics typically inhibit essential cellular processes, including DNA replication, transcription, protein translation and cell wall synthesis (Halawa et al., 2024). These processes impose a significant energy demand on the bacterium, and thus, their disruption leads to imbalance in metabolic homeostasis. Following the interaction between the antibiotic and the primary target, a series of multi-level processes occur downstream that ultimately contribute to bacterial death (Yang et al., 2017; Stokes et al., 2019). In the case of bactericidal antibiotics (*β*-lactams, quinolones or aminoglycosides), bacteria typically exhibit increased tricarboxylic acid cycle (TCA) activity. This results in elevated aerobic respiration rates and the accumulation of reactive oxygen species (ROS), which damage DNA, lipids and proteins (Belenky et al., 2015; Liu et al., 2019).

To mitigate antibiotic-induced stress, bacteria have evolved a variety of defence mechanisms. The main pathway to counteract DNA damage is the SOS response, a coordinated pathway that involves several genes for nucleotide excision repair, error-prone repair synthesis and homologous recombination (Maslowska et al., 2019). The SOS response is activated in the presence of single-strand DNA, which promotes the co-protease activity of RecA. RecA then stimulates the cleavage of the SOS transcriptional repressor LexA, which triggers induction of the SOS regulon. Even at sub-lethal concentrations, bactericidal antibiotics induce DNA damage, thereby inducing the SOS response and eventually increasing bacterial tolerance to antibiotic stress (Shapiro, 2015; Blázquez et al., 2018; Memar et al., 2020). Hence, inhibition of the SOS response has been proposed as an adjuvant strategy to enhance antibiotic efficacy and prevent the evolution of resistance in different bacteria. Various works have reported improvements on bacterial susceptibility to antibiotics by inactivating *recA*, both in laboratory and clinical strains (Recacha et al., 2017; Crane et al., 2021; Machuca et al., 2021; Ledger et al., 2023).

The adaptive response (Ada response) is another important mechanism to counteract DNA damage. This pathway is primarily engaged in the repair of alkylated nucleotides, including N^3^-methyladenine (3meA) and O^6^-methylguanine (O^6^meG) (Mielecki and Grzesiuk, 2014; Mielecki et al., 2015). These base lesions, often generated by environmental alkylating agents but also by products of cellular metabolism, are highly cytotoxic. In *E. coli*, the adaptive response involves four specific proteins for the repair of different types of lesions: Ada (transcriptional activator), AlkB (dioxygenase), AlkA (DNA glycosylase) and AidB (dehydrogenase) (Mielecki and Grzesiuk, 2014; Mielecki et al., 2015). Interestingly, AlkB activity is modulated by the TCA cycle metabolites *α*-ketoglutarate, fumarate and succinate (Silas et al., 2021). Fumarase enzymes catalyse the reversible hydration of fumarate to malate in the TCA cycle and regulate the local concentrations of these metabolites, thereby signaling the DNA damage response. *E. coli* possesses three fumarases: FumA, FumB (class-I fumarases involved in both the TCA cycle and the DNA damage response) and FumC (a class-II fumarase primarily involved in the TCA cycle but capable of mediating the DNA damage response in the absence of other fumarases). Structurally, class-I fumarases are characterized by the presence of a ROS-sensitive catayltic Fe-S cluster, while Class-II fumarases lack this cluster (Woods et al., 1988; Ueda et al., 1991; Flint et al., 1993). Strains lacking these enzymes show compromised survival when exposed to the genotoxic compound methyl methanesulfonate (MMS) (Silas et al., 2021).

In this study, we investigated whether fumarases and the adaptive response also provide protection against antibiotic-induced genotoxicity. Given their roles in the response to DNA damage and central metabolism, we hypothesised that targeting these pathways would also influence the bacterial response to antibiotics. In accordance with this hypothesis, previous authors have reported that fumarase and adaptive response deficiency in *E. coli* leads to enhanced susceptibility to certain antibiotics (Kang et al., 2012; Himpsl et al., 2020). In this study, we initially screened the effect of a large number of antibiotics on *E. coli* BW25113 mutants lacking fumarase (Δ*fumA*, Δ*fumB*, Δ*fumC*) and adaptive response genes (Δ*alkA*, Δ*alkB,* Δ*aidB*). The results showed that inactivation of these genes had a minimal effect on antibiotic susceptibility. The potential of combining the inactivation of these genes with the inactivation of the SOS response was then investigated, since targeting the SOS response in combination with other stress pathways has previously resulted in enhanced sensitisation of the strain BW25113 (Diaz-Diaz et al., 2021, 2022, 2023). Here, double inactivation of the SOS response with either fumarases (Δ*fumC*/Δ*recA*) or else the adaptive response (Δ*aidB*/Δ*recA*) lead to enhanced sensitisation compared to single SOS inactivation, particularly to quinolones and certain *β*-lactams. This phenomenon was more pronounced in the Δ*fumC*/Δ*recA* strain. The findings were assessed in terms of bacterial growth, survival and evolvability. Finally, we further evaluated the impact of *fumC*/*recA* inactivation in a clinical isolate of *E. coli* ST131, where a similar trend towards enhanced antibiotic susceptibility was again observed.

## 2 Materials and methods

### 2.1 Bacterial strains

Wild-type *E. coli* BW25113 and single-gene inactivation mutants (∆*fumA*, ∆*fumB*, ∆*fumC*, ∆*alkA*, ∆a*lkB*, ∆*aidB,* ∆*recA*) were selected from the KEIO collection (Supplementary Table S1) (Baba et al., 2006). Double-gene mutants of *E. coli* BW25113 (∆*fumC/*∆*recA*, ∆*aidB/*∆*recA*) were generated by P1vir phage transduction after removing the kanamycin cassette using plasmid pCP20 (Datsenko and Wanner, 2000; Thomason et al., 2007). The *E. coli* clinical isolate FI20 was provided by the Andalusian Reference Laboratory for Molecular Typing of Nosocomial Pathogens (PIRASOA programme). This isolate belongs to the high-risk clone ST131 and exhibits a low-level quinolone resistance (LLQR) phenotype, as defined by the CLSI reference guidelines (López-Cerero et al., 2013). *E. coli* FI20 single-gene inactivation mutants (∆*fumC*, ∆*recA*) were constructed using a modified version of the Datsenko and Warner method (Datsenko and Wanner, 2000; Machuca et al., 2021) (Supplementary Table S2). In brief, a kanamycin resistance cassette was amplified by PCR using the pKD4 vector as a template and a pair of specific primers containing 5′ extensions homologous to the upstream/downstream sequences of the genes to be inactivated. The primers used were H1-fumC-P1 and H2-fumC-P2 for *fumC* replacement, and H1-recA-P1 and H2-fumC-P2 for *recA* replacement (Supplementary Table S2). The resulting amplicons were used to replace the genomic wild-type genes on the FI20 chromosome by homologous recombination using the Red helper plasmid pKOBEG (Chaveroche et al., 2000), which encodes an arabinose-inducible recombinase. The resulting gene inactivations were confirmed by PCR and Sanger sequencing.

### 2.2 Antimicrobial susceptibility screening

For antimicrobial susceptibility testing of all strains, disc diffusion (Oxoid) was used as the reference method, according to the CLSI guidelines (Performance Standards for Antimicrobial Susceptibility Testing, 34th Edition. CLSI Guideline M100) (CLSI, n.d.). A 0.5 McFarland suspension (10^8^ CFU/mL) of each strain was plated on Mueller-Hinton Agar II (MHA) (Becton, Dickinson and Company), to which relevant antibiotic discs were added. The results were read after overnight incubation at 37°C. The panel of antimicrobial discs consisted of 26 antimicrobial agents and included ampicillin (AMP, 10 μg), amoxicillin (AML, 25 μg), amoxicillin-clavulanic acid (AMC, 30 μg), piperacillin (PRL, 30 μg), piperacillin-tazobactam (TZP, 36 μg), temocillin (TEM, 30 μg), cefepime (FEP, 30 μg), cefotaxime (CTX, 5 μg), ceftazidime (CAZ, 10 μg), ceftazidime-avibactam (CZA, 50 μg), ertapenem (ETP, 10 μg), imipenem (IPM, 10 μg), meropenem (MEM, 10 μg), ciprofloxacin (CIP, 5 μg), levofloxacin (LEV, 5 μg), norfloxacin (NOR, 10 μg), nalidixic acid (NA, 30 μg), amikacin (AK, 30 μg), gentamicin (CN, 10 μg), tobramycin (TOB, 10 μg), chloramphenicol (C, 30 μg), fosfomycin (FOT, 200 μg), rifampicin (RD, 5 μg), trimethoprim (W, 1.25 μg), trimethoprim-sulfamethoxazole (SXT, 25 μg) and tetracycline (TE, 30 μg). Callipers were used to measure the diameter of the inhibition halo (in mm) and to calculate the halo difference between each mutant and its wild-type strain (*E. coli* BW25113 or *E. coli* FI20). The experiment was repeated twice, and the largest halo differences obtained for each antimicrobial/strain combination were noted.

Among the antimicrobials tested by disc diffusion, a *β*-lactam (cefepime) and a quinolone (ciprofloxacin) were selected to determine the Minimum Inhibitory Concentration (MIC) of these antibiotics for all the strains. Gradient strips (Liofilchem) were utilised in accordance with the manufacturer’s instructions. The result was assessed in triplicate for each antimicrobial/strain combination.

### 2.3 Bacterial growth curves

**Figure 1:**
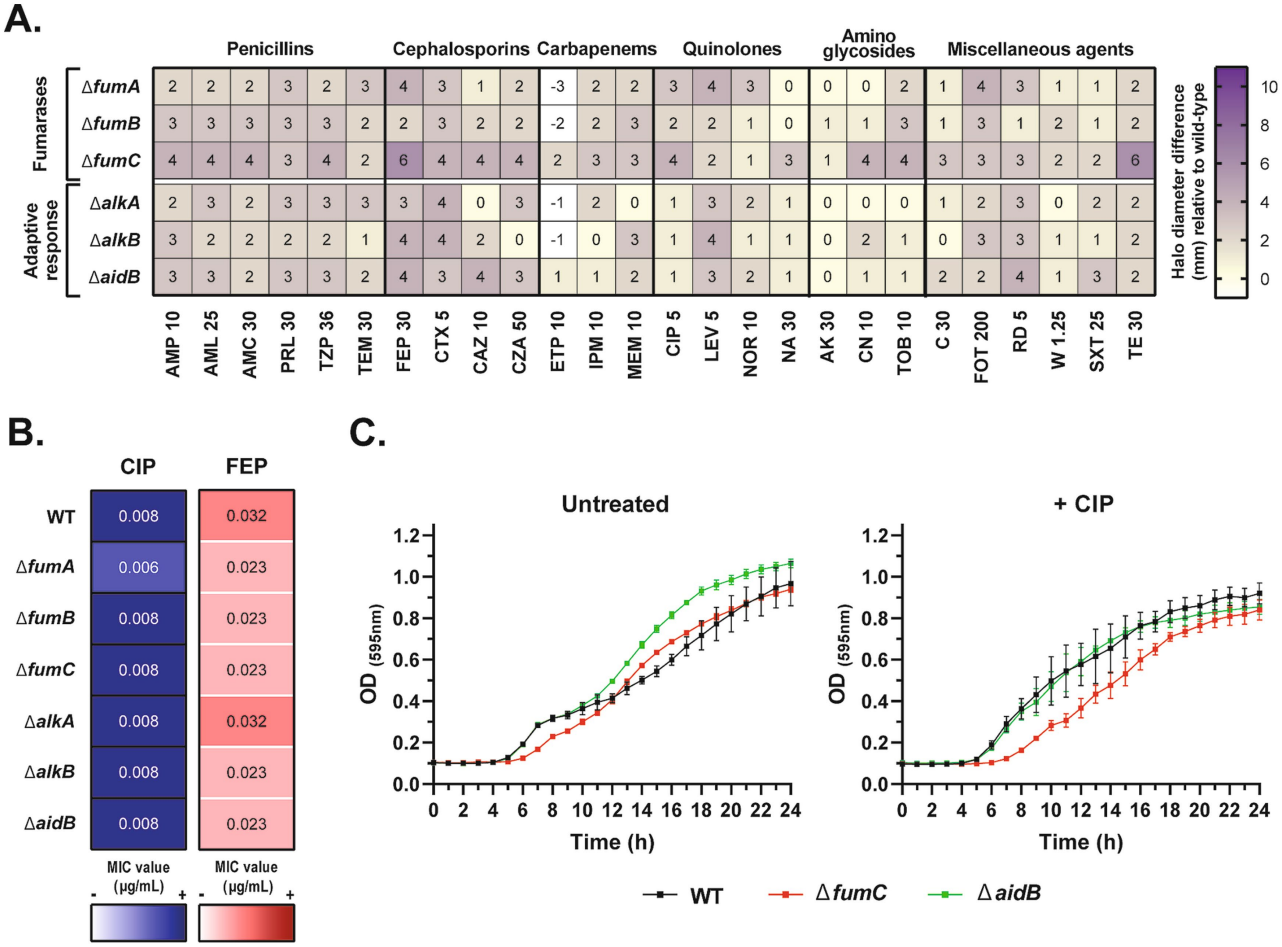
Inactivation of fumarases and the adaptive response resulted in no or slightly increased antimicrobial susceptibility. **(A)** Susceptibility screening by disc diffusion test on *E. coli* BW25113 isogenic mutants with different inactivated fumarases or adaptive response genes. The results are presented as a heatmap showing the differences in inhibition halo diameter (mm) of each mutant versus the BW25113 wild-type (WT) strain. The abbreviations correspond to different antimicrobial discs with the indicated amounts of each (in μg). **(B)** Minimum Inhibitory Concentrations (MIC) of ciprofloxacin (CIP) (blue column) and cefepime (FEP) (red column) determined by E-test for the WT and each BW25113 mutant. Values were determined in triplicate. **(C)** Growth curves of BW25113 WT, Δ*fumC* and Δ*aidB* over 24 h in the absence of antibiotics (*left*) and in the presence of subinhibitory concentrations of ciprofloxacin (0.004 μg/mL, equivalent to 1/2 x MIC of the WT strain) (*right*). Data are the mean of three independent measurements from a representative replicate.

**Figure 2:**
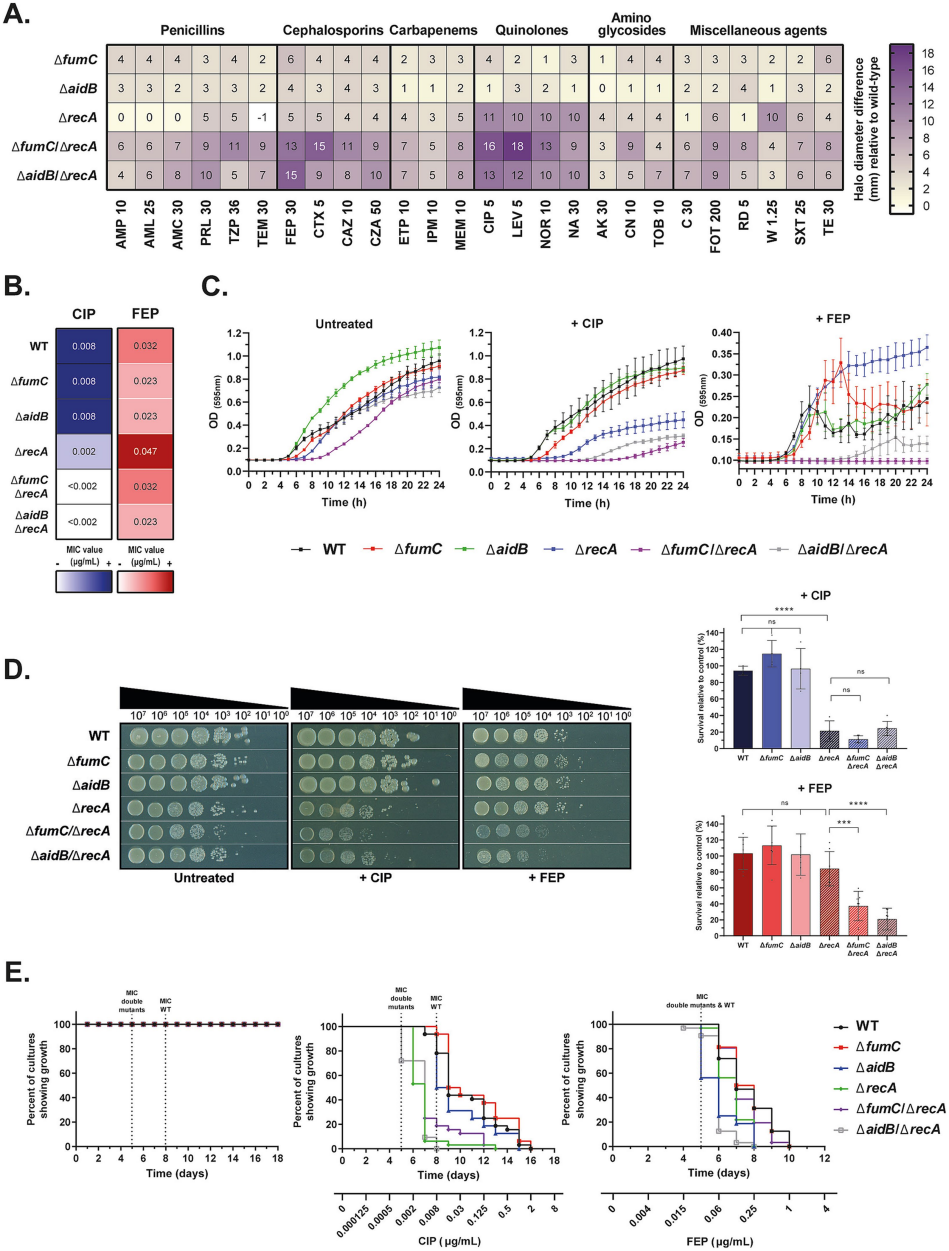
Combined inactivation of the SOS response (*recA*) with fumarase (*fumC*) or the adaptive response (*aidB*) significantly enhances antibiotic susceptibility. **(A)** Susceptibility screening by disc diffusion test on single and double mutants of *E. coli* BW25513. The results are presented as a heatmap, showing the difference in inhibition halo diameter (mm) of each mutant relative to the BW25113 wild-type (WT) strain. The abbreviations correspond to different antimicrobial discs with the indicated amount of each (in μg). **(B)** Minimum Inhibitory Concentrations (MIC) of ciprofloxacin (CIP) (blue column) and cefepime (FEP) (red column) for BW25113 WT and the various mutants by E-test. Values were determined in triplicate. **(C)** Growth curves of all BW25113 strains over 24 h in the absence of antibiotics (*left*), in the presence of subinhibitory concentrations of ciprofloxacin (0.002 μg/mL, equivalent to 1/4 x MIC of the WT strain) (*middle*), and in the presence of subinhibitory concentrations of cefepime (0.016 μg/mL, equivalent to 1/2 x MIC of the WT strain) (*right*). Data are the mean of at least three independent measurements from one representative replicate. **(D)** Survival of BW25113 mutants determined by spot test. A representative replicate of the experiment is shown on the left. Serial dilutions of each strain were spotted on LB agar without antibiotic, or supplemented with ciprofloxacin (0.001 μg/mL, equivalent to 1/8 x MIC of the WT strain) or cefepime (0.008 μg/mL, equivalent to 1/4 x MIC of the WT strain). On the right, mean survival percentage of each mutant under antibiotic pressure (ciprofloxacin above, cefepime below) relative to the untreated control. Data are the mean of at least four independent quantitative measurements. Hatched columns correspond to Δ*recA* mutants. Significant *p* values are recorded (ns, not significant; ***, *p* < 0.001; ****, *p* < 0.0001). **(E)** Evolution capacity of each BW25113 strain without antibiotic pressure (*left*) and in the presence of daily increases of ciprofloxacin (*middle*) and cefepime (*right*) over several days. The dashed vertical line represents the MIC of the WT and the double mutants for each antibiotic.

**Figure 3:**
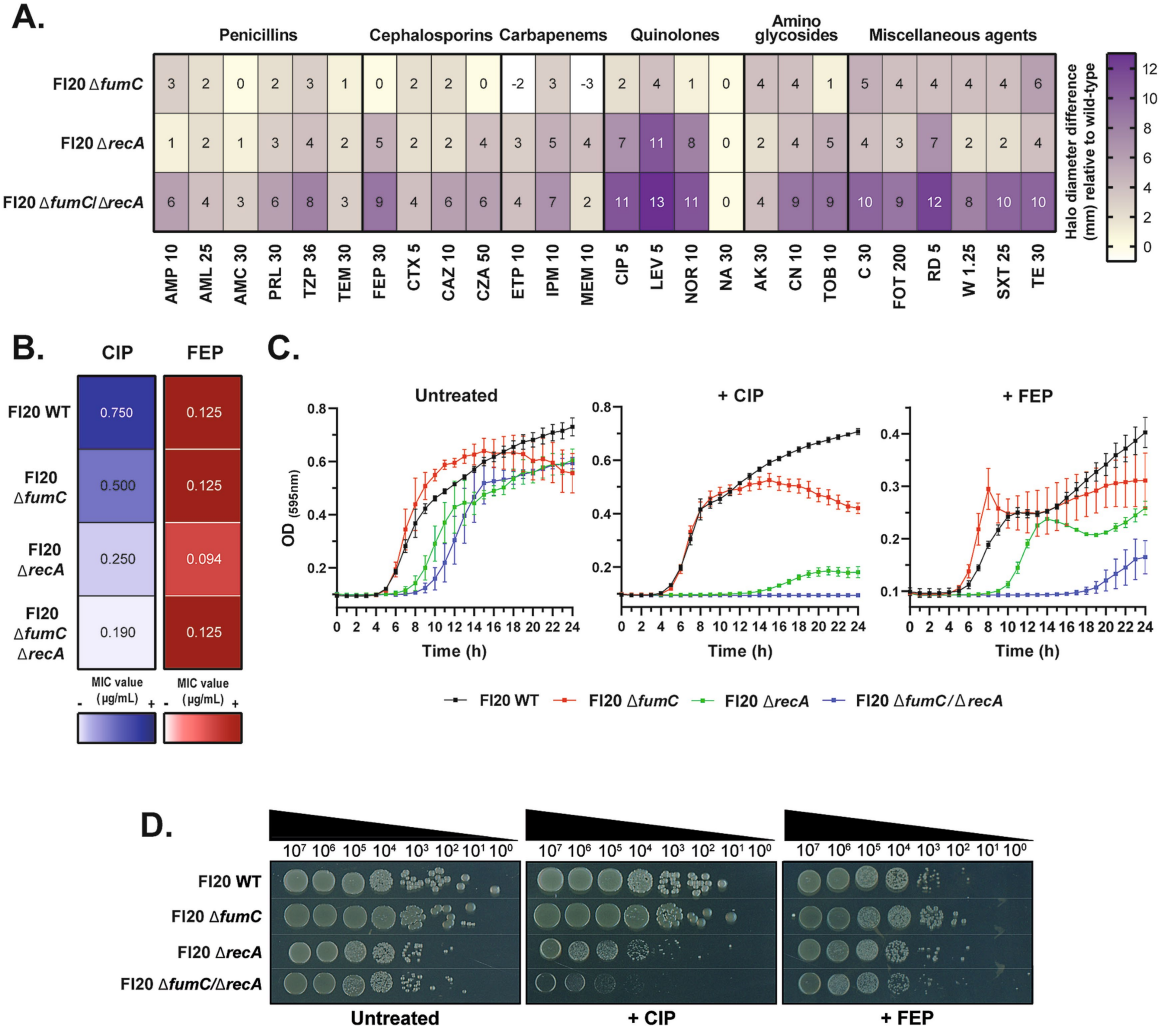
Inactivation of *fumC* and/or *recA* enhances antimicrobial susceptibility in a LLQR ST131 isolate. **(A)** Susceptibility screening by disc diffusion test on the *E. coli* FI20 clinical isolate and its isogenic mutants. The results are displayed as a heatmap, showing the difference in diameter of the inhibition halo (mm) of each mutant versus the FI20 WT strain. The abbreviations correspond to different antimicrobial discs with the indicated amounts (in μg). **(B)** Minimum Inhibitory Concentrations (MIC) of ciprofloxacin (CIP) (blue column) and cefepime (FEP) (red column) for the FI20 WT strain and each mutant by E-test. Values were determined in triplicate. **(C)** Growth curves of all strains over 24 h in the absence of antibiotics (*left*); in the presence of subinhibitory concentrations of ciprofloxacin (0.25 μg/mL, equivalent to 1/3 x MIC of the FI20 WT strain) (*middle*); and in the presence of subinhibitory concentrations of cefepime (0.031 μg/mL, equivalent to 1/4 x MIC of the FI20 WT strain) (*right*). Data are the mean of at least three independent measurements from one representative replicate. **(D)** Survival of FI20 isogenic strains determined by spot test. Serial dilutions of each strain were spotted on LB agar without or with ciprofloxacin (~0.2 μg/mL, equivalent to 1/4 x MIC of the FI20 WT strain) and cefepime (0.031 μg/mL, equivalent to 1/4 x MIC of the FI20 WT strain). A representative replicate of the experiment is shown.

Bacterial growth curves of BW25113 and FI20 were performed to assess the effect of antibiotic pressure in fumarase-, adaptive response- and SOS response-deficient backgrounds. Transparent 96-well flat-bottom plates (Nunclon Delta Surface, Thermo Scientific, MA) were prepared with 200 μL of Luria-Bertani Broth (LBB) (Invitrogen), supplemented with and without sublethal concentrations of ciprofloxacin (Sigma Aldrich) or cefepime (Santa Cruz Biotechnology). Different antibiotic concentrations were used to detect different growth patterns between the WT strain and the isogenic mutants: 0.004 μg/mL CIP (1/2 x MIC of BW25113) in Figure 1C; 0.002 μg/mL CIP (1/4 x MIC of BW25113 WT) or 0.016 μg/mL FEP (1/2 x MIC of BW25113 WT) in Figure 2C; and 0.25 μg/mL CIP (1/3 x MIC of FI20 WT) or 0.031 μg/mL FEP (1/4 x MIC of FI20 WT) in Figure 3C. Next, the LBB 96-well plates were inoculated with 5×10^3^ CFU/mL bacteria previously grown to exponential phase, and bacterial growth was monitored by measuring the OD_595nm_ of each well every hour for 24 h at 37°C using an Infinite200 PRO plate reader (Tecan, Madrid, Spain). Two independent assays with at least three replicates were performed for all conditions evaluated. Finally, the Area Under the Curve (AUC) was calculated using the Growthcurver R package (Sprouffske and Wagner, 2016).

### 2.4 Spot test

Survival of BW25113 and FI20 strains in the presence of antibiotics was evaluated by spot test. 7 μL of serially diluted 0.5 McFarland suspensions (10^8^ CFU/mL) of all strains were spotted on LB Agar (LBA) (Invitrogen) plates containing subinhibitory concentrations of ciprofloxacin or cefepime (1/8 to 1/2 x MIC relative to the wild-type strain of each isogenic group). Only the concentration that produced the most pronounced differences between the strains is shown. After incubation for 20 h at 37°C, the spots were checked for growth and compared with those on a control plate without antimicrobial agent. To quantify bacterial survival, colony counts were performed only when the colonies were large enough to be seen clearly. Bacterial survival was calculated as the percentage of the population that survived in the presence of antibiotics relative to survival in the control without antibiotics. All conditions were tested in at least three independent assays with two replicates each.

### 2.5 Experimental evolution of antibiotic resistance

The ability of *E. coli* BW25113 wild-type and mutant strains to evolve antibiotic resistance was tested using a method that maximises the chances of a population acquiring resistance mutations (Escudero et al., 2018). The experiment compared the behaviour of different strains exposed to gradually increasing concentrations of antibiotics over several days. Briefly, 2 μL of overnight-grown bacteria were inoculated into 96-well plates (Nunclon Delta Surface, Thermo Scientific, MA) containing 198 μL of LBB supplemented with 6.25×10^−5^ μg/mL of ciprofloxacin or 0.001 μg/mL of cefepime. These concentrations corresponded to 1/16 x MIC of the strain with the lowest MIC, determined by the broth microdilution method (data not shown), following to the CLSI guidelines (CLSI, n.d.). After overnight culturing at 37°C without shaking, the turbidity of the wells was quantified by measuring absorbance values at OD_595nm_ using an Infinite200 PRO plate reader (Tecan, Madrid, Spain). Next, 2 μL of the grown culture was transferred to another 96-well plate with a double concentration of antibiotic and incubated again until the following day. This process was repeated daily until OD_595nm_ values were below 0.1, indicating population extinction. Parallel control cultures were performed by propagating the selected strains under identical conditions but without antibiotics. The experiment included a minimum of 16 biological replicates for each strain.

### 2.6 Statistical analysis

All statistical analyses were performed using GraphPad Prism 8 software. Student’s *t-tests* were used to compare two groups. The log-rank (Mantel-Cox) test was used to compare evolution curves in the experimental evolution assay. Differences were considered significant when *p*-values were <0.05.

## 3 Results

### 3.1 Inactivation of fumarases or the adaptive response resulted in null or only slightly increased antimicrobial susceptibility

To identify potential genes involved in antimicrobial sensitisation, we evaluated the response to antimicrobial agents of various *E. coli* BW25113 mutants lacking fumarases (Δ*fumA*, Δ*fumB*, Δ*fumC*) or adaptive response (Δ*alkA*, Δ*alkB*, Δ*aidB*) genes (Supplementary Table S1). As starting point, all mutants were tested by disc diffusion assay for susceptibility to 26 antimicrobials. Compared to the wild-type strain, the mutants exhibited moderate or no change in sensitisation (Figure 1A). Differences in inhibition zone diameter were up to 6 mm, mostly for penicillins, cephalosporins and quinolones. Notably, various mutants were more susceptible to cefepime, with the largest halo difference observed for Δ*fumC* (6 mm). After applying a cut-off of ≥ 3 mm difference, Δ*fumC* was identified as the strain most sensitised to the greatest number of antimicrobial agents, followed by Δ*aidB*. Inhibition zone differences lower than 3 mm were not considered relevant.

To determine the effect of these gene inactivations on MIC, gradient strip assays were performed for a quinolone and a *β*-lactam. Ciprofloxacin and cefepime were selected based on the inhibition zone differences obtained by disc diffusion. No significant changes in susceptibility were observed for either of these antibiotics (Figure 1B). In terms of growth, exposure to 0.004 μg/mL of ciprofloxacin (1/2 x MIC of wild-type BW25113) delayed the growth of Δ*fumC* only slightly, and had no effect on the growth dynamics of Δ*aidB* (Figure 1C, Supplementary Figure S1). Taken together, these data suggest that inactivation of fumarase and adaptive response genes on their own have little influence on antibiotic resistance in *E. coli*.

### 3.2 Combined inactivation of fumarases or the adaptive response together with the SOS response significantly improved overall antimicrobial efficacy

Although targeting fumarases or adaptive response genes alone had little or no effect on antibiotic susceptibility, the hypothesis was considered that simultaneous inactivation of different DNA damage responses would have a greater effect. Therefore, we combined the inactivation of fumarases and adaptive response genes with the inactivation of the SOS response (Δ*recA*). As the *fumC* and *aidB* inactivations showed slightly higher increments of susceptibility (Figure 1A), these genes were selected to construct the double mutant strains: Δ*fumC*/Δ*recA* and Δ*aidB*/Δ*recA* (Supplementary Table S1).

Antibiotic disc diffusion assays were performed to test the phenotype of Δ*fumC*/Δ*recA* and Δ*aidB*/Δ*recA*. As expected, Δ*recA* exhibited reduced resistance to quinolones, with inhibition halo diameters differing by up to 11 mm compared to the wild-type (Figure 2A). Beyond that, the double mutants displayed enhanced susceptibility, particularly to quinolones. The largest differences were observed for ciprofloxacin (16 mm for Δ*fumC*/Δ*recA*; 13 mm for Δ*aidB*/Δ*recA*) and levofloxacin (18 and 12 mm, respectively) (Figure 2A, Supplementary Figure S2). Susceptibility to penicillins and cephalosporins was also significantly increased, particularly for cefepime (13 mm for Δ*fumC*/Δ*recA*; 15 mm for Δ*aidB*/Δ*recA*) and cefotaxime (15 and 9 mm, respectively). It should also be noted that inactivation of *recA* alone did not alter susceptibility to certain antimicrobials, whereas simultaneous inactivation of the pathways did, e.g., amoxicillin–clavulanic acid (7 mm for Δ*fumC*/Δ*recA*; 8 mm for Δ*aidB*/Δ*recA*), temocillin (9 mm for Δ*fumC*/Δ*recA*; 7 mm for Δ*aidB*/Δ*recA*) and chloramphenicol (6 mm for Δ*fumC*/Δ*recA*; 7 mm for Δ*aidB*/Δ*recA*).

To confirm these data, ciprofloxacin and cefepime gradient strip assays were again performed on the mutants. The antimicrobial activity of ciprofloxacin was enhanced against Δ*fumC*/Δ*recA* and Δ*aidB*/Δ*recA*, with a MIC value lower than 0.002 μg/mL. This is a more than two-fold reduction compared to the MIC of 0.002 μg/mL observed against the Δ*recA* mutant (Figure 2B, *left*). However, no reduction in cefepime MICs was detected in the double mutants or in Δ*recA*, compared to the wild-type (Figure 2B, *right*).

### 3.3 Double inactivation of fumarases or the adaptive response together with the SOS response affects bacterial growth and survival under ciprofloxacin and cefepime pressure

To understand the effect of combined inactivation of *fumC*, *aidB* and *recA* under antibiotic exposure, we analysed the ability of double mutants to grow at subinhibitory concentrations of ciprofloxacin and cefepime. In the absence of antibiotics, all *recA*-deficient strains reached similar OD values after 24 h (Figure 2C, *left*; Supplementary Figure S3), although the Δ*fumC*/Δ*recA* mutant exhibited delayed growth during the exponential phase. At a ciprofloxacin concentration of 0.002 μg/mL (1/4 x MIC of wild-type BW25113) (Figure 2C, *middle*), the ODs of all *recA* mutants decreased considerably. This phenomenon was particularly pronounced for Δ*fumC*/Δ*recA* and Δ*aidB*/Δ*recA*. To illustrate, while the mean optical density of Δ*recA* reached 0.41 after 20 h of growth, the OD values of Δ*fumC*/Δ*recA* and Δ*aidB*/Δ*recA* decreased significantly further to 0.16 and 0.25, respectively (*p* < 0.0001) (Supplementary Figure S3). On the other hand, at 0.016 μg/mL of cefepime (1/2 x MIC of wild-type BW25113), all strains showed reduced growth rates. As above, Δ*fumC*/Δ*recA* and Δ*aidB*/Δ*recA* showed markedly diminished growth compared to Δ*recA*, which showed enhanced growth under these conditions (Figure 2C, *right*). While the mean OD of Δ*recA* reached 0.36 after 20 h, the values for Δ*fumC*/Δ*recA* and Δ*aidB*/Δ*recA* were significantly reduced to 0.09 and 0.13, respectively (*p* < 0.0001) (Supplementary Figure S3). AUC comparisons between Δ*recA* and Δ*fumC*/Δ*recA* and Δ*aidB*/Δ*recA* also showed significantly decreased bacterial growth in the presence of the two antibiotics (*p* < 0.05).

Following this line, spot tests were used to determine the survival of the different strains to antibiotics (Figure 2D). Without antibiotic pressure, all strains showed similar survival (Figure 2D*, left*). At 0.001 μg/mL of ciprofloxacin (1/8 x MIC of BW25113 wild-type), survival of wild-type, Δ*fumC* and Δ*aidB* was unaffected, whereas *recA* strains showed increased sensitivity (Figure 2D*, left*). Although a decrease in colony size was observed for Δ*fumC*/Δ*recA*, no significant differences between Δ*recA*, Δ*fumC*/Δ*recA* or Δ*aidB*/Δ*recA* colony counts (*p* > 0.05) were found (Figure 2D, *top right graph*). In contrast, exposure to 0.008 μg/mL of cefepime (1/4 x MIC of BW25113 wild-type) did not affect the survival of Δ*recA*, which was similar to that of the wild-type and the other single mutants (Figure 2D*, left*). Interestingly, in this case, the survival of Δ*fumC*/Δ*recA* and Δ*aidB*/Δ*recA* was negatively affected, in contrast to Δ*recA*. While the mean survival of Δ*recA* was 84%, the percentages for the two double mutants were significantly lower: 37% for Δ*fumC*/Δ*recA* (*p* < 0.001) and 21% for Δ*aidB*/Δ*recA* (*p* < 0.0001) (Figure 2D, *bottom right graph*).

### 3.4 Impact of the inactivation of *fumC*, *aidB* and *recA* in BW25113 on the evolution of acquired resistance to ciprofloxacin and cefepime

*In vitro* evolution experiments were carried out to compare the ability of each strain to acquire resistance in the presence of antibiotics. In these experiments, the concentrations of ciprofloxacin or cefepime were doubled daily to maximise the probability of acquiring resistance mutations. In the absence of antibiotics, no differences between the strains were observed (Figure 2E, *left*).

In the evolution experiment with ciprofloxacin, we started from 6.25×10^−5^ μg/mL. With increasing ciprofloxacin concentration, a general decrease in growth was observed for Δ*recA* strains (no growth at 0.008 μg/mL – 0.25 μg/mL) in contrast to BW25113 wild-type, Δ*fumC* and Δ*aidB* (no growth at 1 μg/mL − 2 μg/mL) (Figure 2E, *middle*). A log-rank test revealed that the differences between these two groups were statistically significant (*p* < 0.0001). However, no significant differences were found when comparing the wild-type with Δ*fumC* or Δ*aidB*, nor between Δ*recA* and Δ*fumC*/Δ*recA* or Δ*aidB*/Δ*recA*. Furthermore, on day 8, when the strains were exposed to the wild-type MIC, the growth capacity of Δ*recA* strains was below 50%, whereas the percentage was the same or higher in BW25113 wild-type, Δ*fumC* and Δ*aidB*.

In the evolution experiment with cefepime, we started from 0.001 μg/mL. With increasing cefepime concentration, all strains survived at concentrations above the MIC of the wild-type strain (0.032 μg/mL), but no growth was detected at concentrations between 0.5 μg/mL and 1 μg/mL (Figure 2E, *right*). As expected, a log-rank test showed significant differences between the wild-type and Δ*recA* (*p* < 0.01). Neither inactivation of *fumC* alone nor combined inactivation with *recA* altered the adaptability of BW25113, whereas a markedly significant difference was found when the wild-type was compared to Δ*aidB* and Δ*aidB*/Δ*recA* (*p* < 0.0001). Significant differences were also found between Δ*recA* and Δ*aidB*/Δ*recA* (*p* < 0.001), indicating that the impact of *aidB* inactivation can be enhanced by also inactivating the SOS response.

### 3.5 Combined inactivation of *fumC* and *recA* substantially restores the susceptibility of a high-risk clone with low-level clinical resistance

Since joint inactivation of *fumC*, *aidB* and *recA* showed increased sensitisation in a laboratory strain of *E. coli*, we decided to test this strategy on the FI20 clinical isolate (ST131 clone), which is a strain with low-level resistance to quinolones (LLQR). Having observed that the BW25113 Δ*fumC*/Δ*recA* mutant was slightly more susceptible to antibiotics than Δ*aidB*/Δ*recA*, we focused on the first combination and generated FI20 Δ*fumC*, Δ*recA* and Δ*fumC*/Δ*recA* mutants (Supplementary Table S1).

As above, disc diffusion screening tests were performed on the *E. coli* FI20 strains (Figure 3A). FI20 Δ*fumC* showed a maximum halo difference of 6 mm, especially for aminoglycosides and miscellaneous agents. FI20 Δ*recA* displayed increased sensitisation to quinolones. Remarkably, FI20 Δ*fumC*/Δ*recA* exhibited larger zones of inhibition than FI20 Δ*recA*. The major differences were for ampicillin (6 mm difference in FI20 Δ*fumC*/Δ*recA*; 1 mm in FI20 Δ*recA*), cefepime (9 mm in FI20 Δ*fumC*/Δ*recA*; 5 mm in FI20 Δ*recA*), ciprofloxacin (11 mm in FI20 Δ*fumC*/Δ*recA*; 7 mm in FI20 Δ*recA*), fosfomycin (9 mm in FI20 Δ*fumC*/Δ*recA*; 3 mm in FI20 Δ*recA*) and trimethoprim (8 mm in FI20 Δ*fumC*/Δ*recA*; 2 mm in FI20 Δ*recA*) (Figure 3A, Supplementary Figure S4), among others. We then assessed these results by E-test and observed a gradual decrease in ciprofloxacin MICs from FI20 Δ*fumC* and FI20 Δ*recA* to FI20 Δ*fumC*/Δ*recA* (Figure 3B, *left*). In contrast, no differences in cefepime MICs were observed, except for a slight decrease in F120 Δ*recA* (Figure 3B, *right*).

With respect to bacterial growth, all strains in the untreated controls behaved in a similar way (Figure 3C, *left*). At a ciprofloxacin concentration of 0.25 μg/mL (1/3 x MIC of wild-type FI20), only FI20 Δ*fumC*/Δ*recA* growth was completely inhibited (Figure 3C, *middle*). After 20 h of growth, the mean ODs of FI20, FI20 Δ*fumC*, FI20 Δ*recA* and FI20 Δ*fumC*/Δ*recA* were 0.71, 0.45, 0.29 and 0.10, respectively (Supplementary Figure S5). Significant differences were found in the ODs (*p* < 0.01) and AUCs (*p* < 0.001) of FI20 Δ*recA* and FI20 Δ*fumC*/Δ*recA*. At a cefepime concentration of 0.031 μg/mL (1/4 x MIC of wild-type FI20), growth of FI20 Δ*recA* and FI20 Δ*fumC*/Δ*recA* was significantly impaired (Figure 3C, *right*). After 20 h, the ODs of FI20, FI20 Δ*fumC*, FI20 Δ*recA* and FI20 Δ*fumC*/Δ*recA* were 0.37, 0.28, 0.25 and 0.14, respectively (Supplementary Figure S5). Similarly, significant differences were found in the ODs (*p* < 0.01) and AUCs (p < 0.001) of FI20 Δ*recA* and FI20 Δ*fumC*/Δ*recA*.

Survival of the FI20 strains was also evaluated by spot test (Figure 3D). Following exposure to 0.2 μg/mL ciprofloxacin (1/4 x MIC of wild-type FI20), the strains with inactivated SOS response were more susceptible than FI20 wild-type and FI20 Δ*fumC*. Moreover, FI20 Δ*fumC*/Δ*recA* was even more sensitised than the single FI20 Δ*recA* mutant (about 10^1^-fold). However, exposure to 0.031 μg/mL cefepime (1/4 x MIC of wild-type FI20) did not significantly alter the survival of FI20 Δ*fumC*/Δ*recA* compared to FI20 Δ*recA* or FI20 Δ*fumC*.

## 4 Discussion

Stress response mechanisms allow bacteria to adapt to and survive antibiotic pressure. These mechanisms therefore represent potential molecular targets for enhancing antibiotic activity and slowing the development of antibiotic resistance (Dawan and Ahn, 2022). The aim of this study was to investigate whether a recently identified pathway that protects against genotoxic damage also provides protection against antibiotic stress (Silas et al., 2021). This pathway involves the interplay between fumarases (*fumA, fumB, fumC*) and the adaptive response (*alkA*, *alkB*, *aidB*). The absence of fumarases and the adaptive response is associated with the inability of bacteria to survive in the presence of MMS, a compound that methylates DNA bases, blocking the progression of DNA polymerase or promoting the potential accumulation of miscoding nucleotides (Mielecki et al., 2015). Some antibiotics also induce DNA damage and increase genomic instability (Shapiro, 2015). For instance, quinolones generate double-strand DNA breaks by inhibiting DNA gyrase and topoisomerase (Drlica, 1999), while other antibiotics damage DNA indirectly by increasing ROS levels, disrupting the balance of nucleotide pools or interfering with the correct translation of proteins (Blázquez et al., 2012). Taken together, these factors led us to hypothesise that the inactivation of fumarase and the adaptive response could also help to make bacteria more susceptible to antibiotics.

Here, we first screened the impact of multiple antimicrobial agents on inactivated strains of *E. coli* BW25113 Δ*fumA*, Δ*fumB*, Δ*fumC* (fumarases) and Δ*alkA*, Δ*alkB* and Δ*aidB* (adaptive response) (Figure 1, Supplementary Figure S1). These inactivations had no or very little effect on antibiotic susceptibility. Similar results have previously been described in *E. coli* CFT073 Δ*fumA*, Δ*fumB*, Δ*fumC* mutants for chloramphenicol, trimethoprim, tetracycline, ciprofloxacin, ampicillin and streptomycin, although the latter demonstrated an elevated MIC for Δ*fumC* (Himpsl et al., 2020). It is also noteworthy that *fumC* deficiency in *Staphylococcus aureus* results in higher tolerance to ciprofloxacin, gentamycin and oxacillin due to reduced ATP intracellular levels (Zalis et al., 2019). Of the inactivations targeted in our study, those of *fumC* and *aidB* had the greatest effect, particularly against penicillins and cephalosporins. FumC is a class II fumarase, that is, a fumarase without a catalytic Fe-S cluster, as opposed to the class I fumarases FumA and FumB, which do contain this cluster (Woods et al., 1988; Ueda et al., 1991). Fe-S clusters are typically oxidised in the presence of ROS (Flint et al., 1993). Consequently, in the presence of antibiotics that induce oxidative stress, FumA and FumB may lose their catalytic activity, leaving only FumC as the active fumarase. Previous studies have shown that in the absence of FumA and FumB, FumC participates in the response to DNA damage and TCA cycle functions (Silas et al., 2021). In the context of a Δ*fumC* mutant, the absence of FumA and FumB would mean the lack of fumarase activity during antibiotic-induced oxidative stress. This could be one explanation for the increased susceptibility observed in Δ*fumC* compared to the other mutants. In addition, inactivation of fumarases in the TCA cycle may also trigger metabolic perturbations that also affect antibiotic susceptibility. For example, fumarase deficiency in *Mycobacterium tuberculosis* is bactericidal due to an accumulation of fumarate, which intereferes with catalase and mycothiol antioxidants, ultimately leading to oxidative stress (Ruecker et al., 2017). In this sense, fumarase inhibitors have been developed for this bacterium in the search for novel antibacterial compounds (Whitehouse et al., 2019).

Regarding the adaptive response, it is already known that inactivating *alkA* and *alkB* leads to increased susceptibility to kanamycin, while *alkB* inactivation does not affect survival to ciprofloxacin (Kang et al., 2012). In our screening with multiple antimicrobial agents, we only detected small or negligible increments in the susceptibility of Δ*alkA*, Δ*alkB* and Δ*aidB*. AidB is a component of the adaptive response, and its function is as yet unclear. It shows homology to acyl-CoA oxidases and has been reported to bind to double-strand DNA for dealkylation (Mielecki and Grzesiuk, 2014). Of note, inactivation of *aidB* in *E. coli* does not appear to affect bacterial survival in the presence of MMS (Rippa et al., 2011). Previous studies have shown that Ada, the transcriptional regulator of the adaptive response, is induced in the presence of MMS and subinhibitory concentrations of aminoglycosides, *β*-lactams and quinolones (Gutierrez et al., 2013), suggesting that this mechanism may be involved in the antibiotic-induced stress response. Indeed, *ada* is regulated by the general RpoS stress response regulon, which enables bacteria to combat antibiotic stress induced by metabolic disturbances and ROS production (Weber et al., 2005; Blázquez et al., 2018). This may explain why we observed some increases in antibiotic susceptibility in the absence of adaptive response genes.

Given the modest differences obtained, we decided to inactivate fumarases and the adaptive response together with the SOS response. Targeting the SOS response through *recA* inactivation has been shown to reverse quinolone resistance *in vitro* and *in vivo* (Recacha et al., 2017; Machuca et al., 2021). Furthermore, the combined inactivation of *recA* and other anti-genotoxic stress pathways, such as the ROS detoxification systems (*sodB, katG*) or Dam methylase, resulted in an enhanced or even synergistic increase in susceptibility (Diaz-Diaz et al., 2021, 2023). In this study, attending to the disc difussion results (Figure 2, Supplementary Figures S2, S3), we observed that strains doubly defective for *fumC*/*recA* or *aidB*/*recA* tended to be increasingly susceptible to various antimicrobial agents (Figure 2, Supplementary Figures S2, S3). This effect was particularly marked for β-lactams and was not found after inactivation of Δ*recA* alone. In the presence of a quinolone (ciprofloxacin) and a β-lactam (cefepime), the double mutants showed delayed or no growth. In terms of survival and the ability to evolve resistance, cefepime activity was potentiated by double inactivations, particularly Δ*aidB*/Δ*recA*. These results are consistent with the reduced survival of a Δ*lexA3*/Δ*ada* mutant following continuous exposure to MMS (Uphoff, 2018). However, in the presence of ciprofloxacin, double inactivations had no effect on survival or capacity to evolve compared to Δ*recA.* This can also be seen as a positive outcome, as in previous studies carried out in our laboratory, targeting the SOS response together with ROS detoxification systems improved evolvability compared to single Δ*recA* inactivation (Diaz-Diaz et al., 2022).

It is likely that the lack of two distinct DNA damage responses makes it more difficult for bacteria to overcome the side effects of antibiotic-induced stress. Although the SOS response and the adaptive response act on gene damage, the outcome of their activity is very different. The adaptive response involves the faithful repair of alkylation damage, whereas the SOS response activates low-fidelity polymerases that increase the rate of mutagenesis and thus the likelihood of resistance emergence beyond DNA repair. Similarly, the SOS response to MMS is activated more rapidly than the adaptive response, so that the inactivation of both pathways would prevent DNA repair for a longer period of time (Uphoff, 2018; Kamat and Badrinarayanan, 2023). It is also worth mentioning that the activity of the SOS response in *Bacillus subtilis* depends partly on the activity of the fumarase Fum-bc, which is recruited to DNA double-strand breaks produced by MMS. Through the production of L-malate, Fum-bc upregulates the local translation of RecN, one of the first proteins recruited to DNA damage sites during the SOS response to promote repair (Alonso et al., 2013; Singer et al., 2017; Leshets et al., 2018). The absence of Fum-bc would also presumably delay RecN-dependent repair. In other words, all these pathways are also interconnected, which explains why targeting them together has a greater effect on sensitisation.

Since the inactivation of fumarases, the adaptive response and the SOS response enhanced antimicrobial activity in a susceptible *E. coli* strain, we assessed these results in a strain with intrinsic resistance mechanisms. The FI20 clinical isolate was selected to reproduce these experiments (Figure 3, Supplementary Figures S4, S5). This strain belongs to the high-risk ST131 clone and contains mutations in *gyrB* and *parC* that confer quinolone resistance (Hernandez et al., 2011; Machuca et al., 2021). Since the BW25113 Δ*fumC*/Δ*recA* mutant showed slightly higher differences on susceptibility for various antimicrobial agents than BW25113 Δ*aidB*/Δ*recA*, it was decided to focus on the first strategy and to generate a FI20 Δ*fumC*/Δ*recA* mutant. As with the BW25113 strain, single inactivation of Δ*fumC* resulted in very modest differences in susceptibility, in this case, to miscellaneous agents. Interestingly, Δ*fumC* deficiency in FI20 also had a negative effect on growth under antibiotic exposure, in contrast to the susceptible BW25113 strain. With respect to the FI20 Δ*fumC*/Δ*recA* mutant, we again observed a marked increase in susceptibility, which was significant mainly for quinolones and miscellaneous agents such as rifampicin. This suggests that in addition to the higher sensitisation achieved by *recA* inactivation in BW25113, this strategy further enhances quinolone efficacy in a resistant strain. This is consistent with the results obtained from growth curves and spot tests.

In general, the results of this study indicate that the differences between the various techniques used were not always equitable and that the gene inactivations evaluated did not consistently result in high fold-reductions in MIC values. Nevertheless, we showed that there were significant changes in antibiotic susceptibility at the biological level and using different approaches. It is important to highlight that the correlation between MIC and pharmacodynamic parameters is not always exact (Rodríguez-Martínez et al., 2016). In the case of the FI20 clinical isolate, growth curves and spot tests showed that, when fumarase and the *recA*-dependent SOS response were absent, moderate concentrations of antibiotic had a marked effect on the susceptibility of this LLQR bacterium, suggesting that this strategy may have potential clinical applicability in the treatment of this resistant phenotype.

Overall, the present study shows that combined inactivation of fumarase and the SOS response is a potential sensitisation strategy that has not been previously considered or characterised. It also shows that this phenomenon applies to antimicrobials and not only to alkylating agents, and suggests ways for new therapeutic strategies to combat antimicrobial resistance and enhance antibiotic activity.

## Data Availability

The datasets presented in this study can be found in online repositories. The names of the repository/repositories and accession number(s) can be found below: https://www.ncbi.nlm.nih.gov/, PRJNA1015411.
