# Supplementary material for: Combined inactivation of the SOS response with TCA fumarases and the adaptive response enhances antibiotic susceptibility against Escherichia coli
Source: Front Microbiol. 2025 May 9;16:1570764. doi: 10.3389/fmicb.2025.1570764 (PMC12098349; doi:10.3389/fmicb.2025.1570764)
Supplement: Supplementary file 1 [file Data_sheet_1.DOCX]

Supplementary Material

# Table S1. Strains used in this study.

| **Strain** | **Genotype** | **Pathway(s) affected** | | **Mechanism of quinolone resistance**^c^ | | | | **Source** |
| --- | --- | --- | --- | --- | --- | --- | --- | --- |
|  |  |  |  | ***gyrA*** | ***gyrB*** | ***parC*** | ***parE*** |  |
| *E. coli* BW25113^a^ | wild-type *E. coli* strain BW25113 | - | | - | - | - | - | (Baba et al., 2006) |
| *E. coli* BW25113 ∆*fumA*^a^ | *fumA* knock-out | TCA cycle and DNA repair signalling | | - | - | - | - | (Baba et al., 2006) |
| *E. coli* BW25113 ∆*fumB*^a^ | *fumB* knock-out |  |  | - | - | - | - | (Baba et al., 2006) |
| *E. coli* BW25113 ∆*fumC*^a^ | *fumC* knock-out |  |  | - | - | - | - | (Baba et al., 2006) |
| *E. coli* BW25113 ∆*alkA*^a^ | *alkA* knock-out | Adaptive response | | - | - | - | - | (Baba et al., 2006) |
| *E. coli* BW25113 ∆*alkB*^a^ | *alkB* knock-out |  |  | - | - | - | - | (Baba et al., 2006) |
| *E. coli* BW25113 ∆*aidB*^a^ | *aidB* knock-out |  |  | - | - | - | - | (Baba et al., 2006) |
| *E. coli* BW25113 ∆*recA*^a^ | *recA* knock-out | SOS response | | - | - | - | - | (Baba et al., 2006) |
| *E. coli* BW25113 ∆*fumC*/∆*recA*^a^ | *fumC / recA* double knock-out | TCA cycle and DNA repair signalling, SOS response | | - | - | - | - | This study |
| *E. coli* BW25113 ∆*aidB*/∆*recA*^a^ | *aidB / recA* double knock-out | Adaptive and SOS responses | | - | - | - | - | This study |
| *E. coli* FI20^b^ | *E. coli* clinical strain FI20 | - | | S83L | - | S80R | I529L | (Machuca et al., 2021) |
| *E. coli* FI20 ∆*fumC*^b^ | *fumC* knock-out | TCA cycle and DNA repair signalling | | S83L | - | S80R | I529L | This study |
| *E. coli* FI20 ∆*recA*^b^ | *recA* knock-out | SOS response | | S83L | - | S80R | I529L | (Machuca et al., 2021) |
| *E. coli* FI20 ∆*fumC*/∆*recA*^b^ | *fumC* / *recA* double knock-out | TCA cycle and DNA repair signalling, SOS response | | S83L | - | S80R | I529L | This study |
| ^a^ Isogenic strains of BW25113 carrying only the inactivated gene(s) indicated. | | |  | | | | | |
| ^b^ Isogenic strains of clinical isolate F120 carrying only the inactivated gene(s) indicated. FI20 is an ST131 clone according to the University of Warwick MLST scheme (https://enterobase.warwick.ac.uk | | | | | | | | |
| ^c^ Resistance-associated mutations in *gyr* and *par* genes have been identified as quinolone-resistance determinants. Aminoacid changes are shown. | | | | | | | | |

# Table S2. Oligonucleotides, phages and plasmids used in this study for genetic manipulation.

| **Oligonucleotides** | | | |
| --- | --- | --- | --- |
| **Oligonucleotides for gene inactivation testing** | | | |
| **Name** | **Sequence (5' -> 3')** | **Description and annealing location** | **Reference** |
| pre-fumA | TGGAGCCGCAAAAAGTCGTA | *fumA* locus-specific, forward and external | This study |
| post-fumA | ATCTGCCGGGACATCAATCG | *fumA* locus-specific, reverse and external | This study |
| pre-fumB | CCGCGATGTACGGGTTCTTA | *fumB* locus-specific, forward and external | This study |
| post-fumB | TGAGTTTGTCATGGGCCGAA | *fumB* locus-specific, reverse and external | This study |
| pre-fumC | TGCACCCGCTGTGTGAAATA | *fumC* locus-specific, forward and external | This study |
| post-fumC | TGCCCTACACCACTGATTGC | *fumC* locus-specific, reverse and external | This study |
| pre-alkA | GACCGCCACTGAACAGTTTG | *alkA* locus-specific, forward and external | This study |
| post-alkA | TTAACTGAGGCGTGCTTCCC | *alkA* locus-specific, reverse and external | This study |
| pre-alkB | TGCGGTGAAACCGTCAGTTA | *alkB* locus-specific, forward and external | This study |
| post-alkB | TTCCATGCAGAAAACCCGGA | *alkB* locus-specific, reverse and external | This study |
| pre-aidB | CTCACGTTTCGTTCCGCATT | *aidB* locus-specific, forward and external | This study |
| post-aidB | TGATAACTGGCGCGTACAGG | *aidB* locus-specific, reverse and external | This study |
| pre-recA | TCGTCAGGCTACTGCGTATGCAT | *recA* locus-specific, forward and external | (Recacha et al., 2017) |
| post-recA | GTACCGCACGATCCAACAGGCGA | *recA* locus-specific, reverse and external | (Recacha et al., 2017) |
| K1 | CAGTCATAGCCGAATAGCCT | Kanamycin cassette-specific, reverse and internal | (Datsenko and Wanner, 2000) |
| K2 | CGGTGCCCTGAATGAACTGC | Kanamycin cassette-specific, forward and internal | (Datsenko and Wanner, 2000) |

| **Oligonucleotides using the Datsenko inactivation protocol** | | |
| --- | --- | --- |
| **Sequence (5' -> 3')**^a^ | **Description** | **Reference** |
| GTTGCTTAACGAAAGCAAAACAGAAAGAAAAAATTAATCAGGTGAGGAGCAGGTCATGAATACAGTACGCgtgtaggctggagctgcttc | Forward primer for *fumC* inactivation, with a 5’-extension homologous to the upstream *fumC* genomic region | This study |
| ACCTCAGGCGCAGCCGCTTCGTTTGATCATTCCACGGCTGCACCTGTATGTTGCAGATTAACGCCCGGCTTTatgggaattagccatggtcc | Reverse primer for *fumC* inactivation, with a 5’-extension homologous to the downstream *fumC* genomic region | This study |
| CAGAACATATTGACTATCCGGTATTACCCGGCATGACAGGAGTAAAAATGGCTATCGACGAAAACAAACAgtgtaggctggagctgcttc | Forward primer for *recA* inactivation, with a 5’-extension homologous to the upstream *recA* genomic region | (Recacha et al., 2017) |
| ATGCGACCCTTGTGTATCAAACAAGACGATTAAAAATCTTCGTTAGTTTCTGCTACGCCTTCGCTATCATatgggaattagccatggtcc | Reverse primer for *recA* inactivation, with a 5’-extension homologous to the downstream *recA* genomic region | (Recacha et al., 2017) |

| **Phage** | |  |
| --- | --- | --- |
| **Description** | **Reference** |  |
| Gene inactivation by P1 phage transduction | (Thomason et al., 2007) |  |
|  | |  |
| **Plasmids** | |  |
| **Description** | **Reference** |  |
| Plasmid encoding a FLP heat shock exonuclease. Used for excision of the kanamycin resistance cassette from the chromosome via FRT sites. | (Datsenko and Wanner, 2000) |  |
| Plasmid used as PCR template to amplify the kanamycin resistance gene and flanking FRT sites for chromosomal inactivation. | (Datsenko and Wanner, 2000) |  |
| Plasmid encoding an arabinose-inducible λ-red recombinase and a chloramphenicol resistance marker. Contains a temperature-sensitive origin of replication. Used for chromosomal gene replacement. | (Chaveroche et al., 2000) |  |
| ^a^  Gene flanking regions are indicated in capital letters; the start/stop codon is underlined; the region annealing with the kanamycin cassette is indicated in small letters | | |


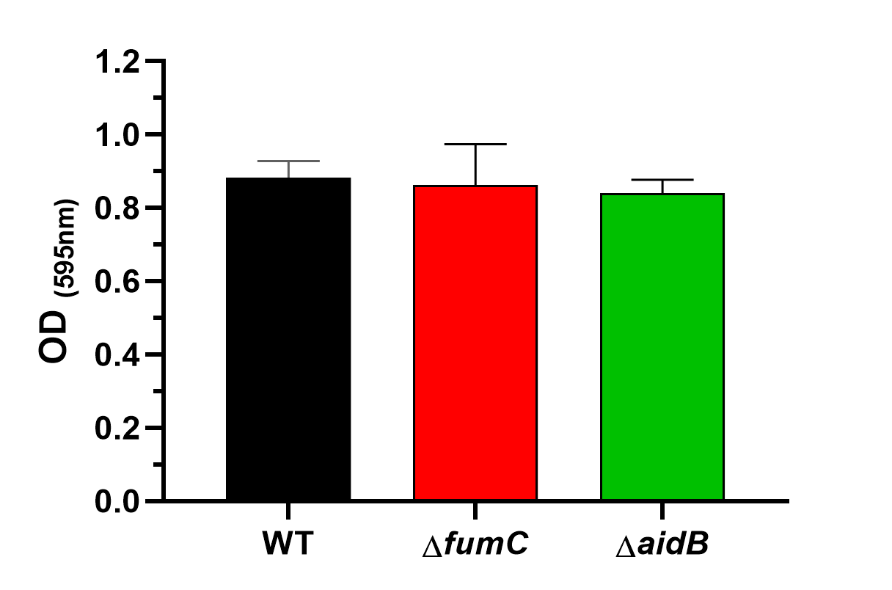


**Fig S1. Effect of fumarases and adaptive response genes on the growth of *E. coli* BW25113**. OD_595nm_ values of BW25113 WT, Δ*fumC* and Δ*aidB* after 20 h of growth in LBB culture supplemented with 0.004 μg/mL ciprofloxacin (equivalent to 1/2 x MIC of the WT strain). Data are the mean of six independent measurements.


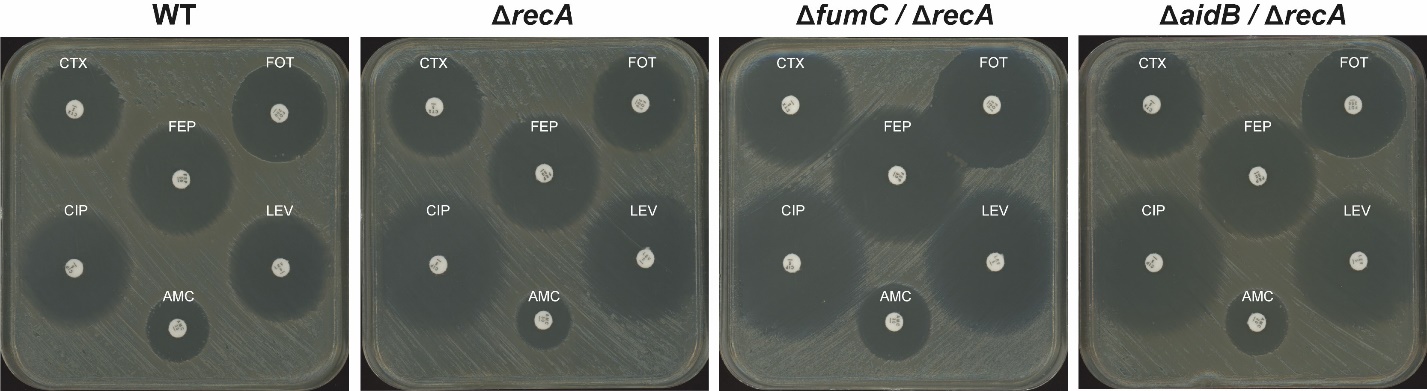


**Fig S2. Enhanced antibiotic sensitisation of *E. coli* BW25113 by targeting the SOS response together with fumarases or the adaptive response.** Representative experiment of disc diffusion assays performed on single and double mutants of BW25113. The antibiogram shows the antimicrobials that produced the largest differences in inhibition halo diameters for the double mutants, including cefotaxime (CTX), fosfomycin (FOT), cefepime (FEP), ciprofloxacin (CIP), levofloxacin (LEV) and amoxicillin-clavulanic acid (AMC).


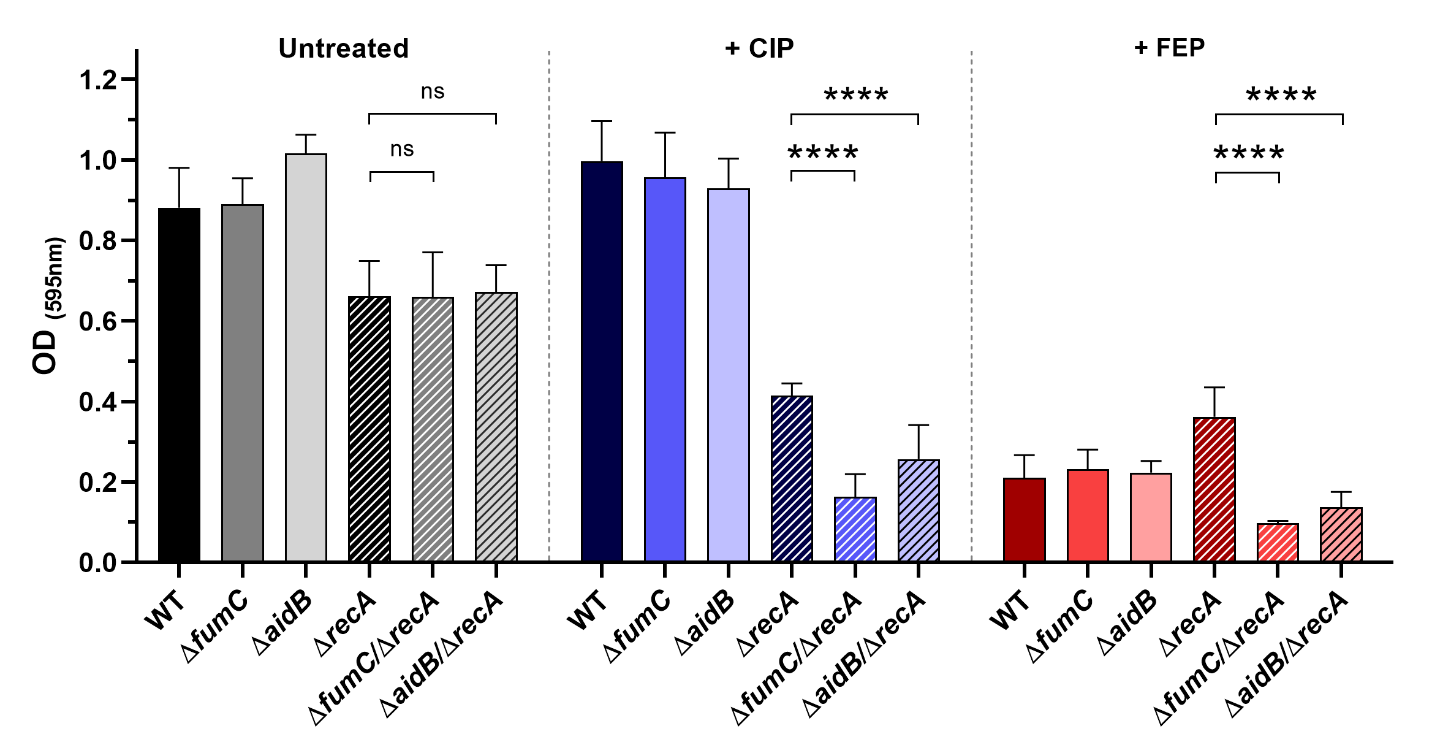


**Fig S3. Effect of single and double inactivation of *fumC, aidB* and *recA* on the growth of *E. coli* BW25113**. OD_595nm_ values of all strains after 20 h of growth in LBB cultures without antibiotics (black columns), supplemented with ciprofloxacin (0.002 μg/mL, equivalent to 1/4 x MIC of the WT strain) (blue columns) or with cefepime (0.016 μg/mL, equivalent to 1/2 x MIC of the WT strain) (red columns). Hatched columns correspond to Δ*recA* mutants. Data are the mean of ten independent measurements from at least two different experiments. Significant *P* values are indicated (ns, not significant; ****, *P* < 0.0001).


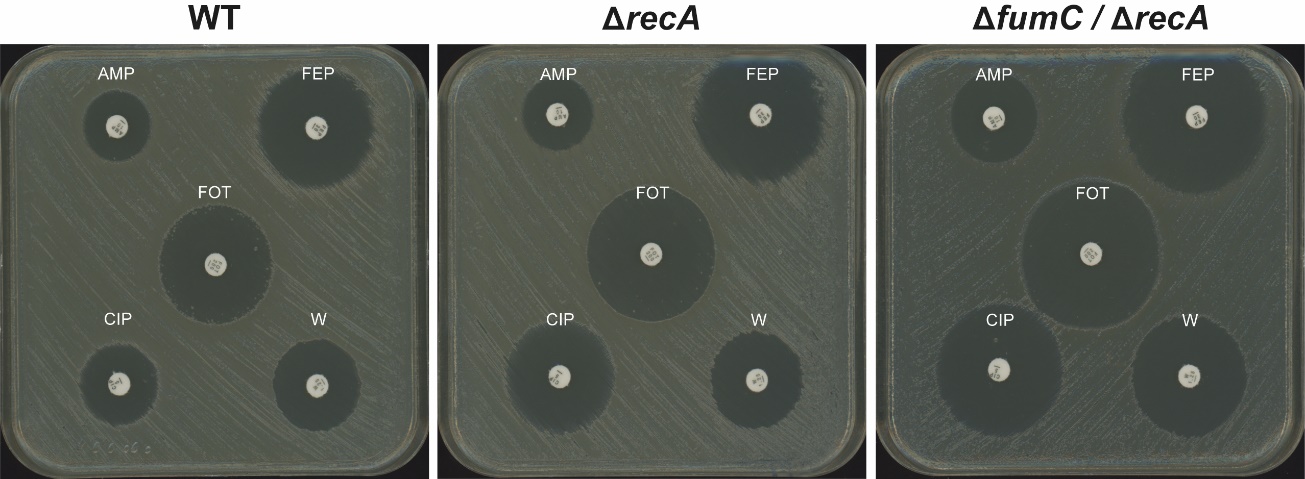


**Fig S4. Enhanced antibiotic sensitisation of *E. coli* FI20 by targeting *recA* together with *fumC*.** Representative disc diffusion assay on single- and double-inactivation mutants of F120. The antibiogram shows the antimicrobials that produced the largest differences in inhibition zone diameters against the double mutant, including ampicillin (AMP), cefepime (FEP), fosfomycin (FOT), ciprofloxacin (CIP) and trimethoprim (W).


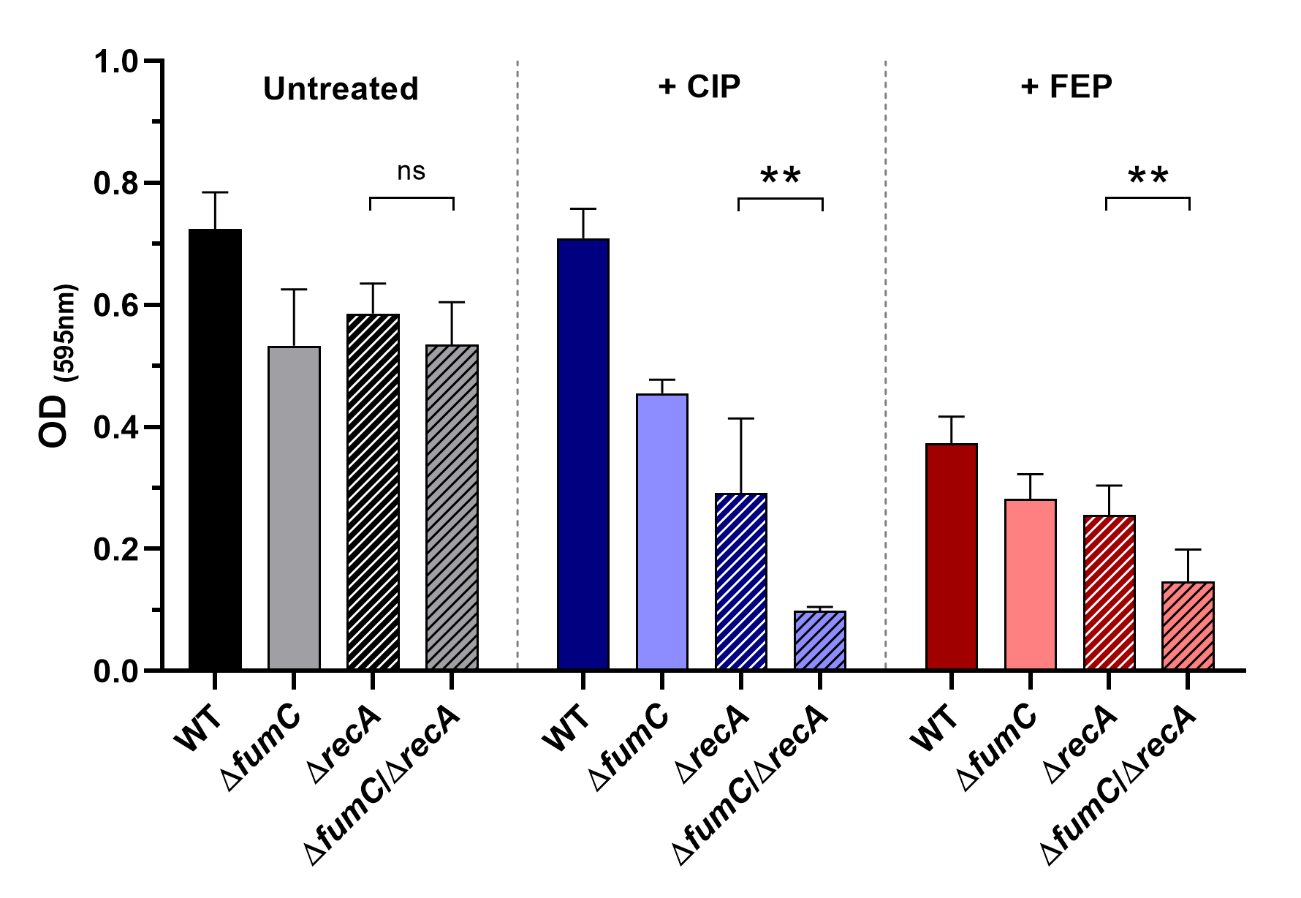


**Fig S5. Effect of *fumC* and/or *recA* inactivations on growth of FI20**. OD_595nm_ values of all strains after 20 h of growth in LBB cultures: without antibiotics (black columns), supplemented with ciprofloxacin (0.25 μg/mL, equivalent to 1/3x MIC of the WT FI20 strain) (blue columns) or cefepime (0.031 μg/mL, equivalent to 1/4 x MIC of the WT FI20 strain (red columns). Hatched columns represent Δ*recA* mutants. Data are the mean of ten independent measurements from at least two different experiments. Significant *P* values are indicated (ns, not significant; **, *P* < 0.01).

**Supplementary material bibliography**

Baba, T., Ara, T., Hasegawa, M., Takai, Y., Okumura, Y., Baba, M., et al. (2006). Construction of *Escherichia coli* K‐12 in‐frame, single‐gene knockout mutants: the Keio collection. *Molecular Systems Biology* 2, 2006.0008. doi: 10.1038/msb4100050

Chaveroche, M.-K., Ghigo, J.-M., and d’Enfert, C. (2000). A rapid method for efficient gene replacement in the filamentous fungus *Aspergillus nidulans*. *Nucleic Acids Research* 28, e97. doi: 10.1093/nar/28.22.e97

Datsenko, K. A., and Wanner, B. L. (2000). One-step inactivation of chromosomal genes in *Escherichia coli* K-12 using PCR products. *Proceedings of the National Academy of Sciences* 97, 6640–6645. doi: 10.1073/pnas.120163297

Machuca, J., Recacha, E., Gallego-Mesa, B., Diaz-Diaz, S., Rojas-Granado, G., Garciá-Duque, A., et al. (2021). Effect of RecA inactivation on quinolone susceptibility and the evolution of resistance in clinical isolates of *Escherichia coli*. *Journal of Antimicrobial Chemotherapy* 76, 338–344. doi: 10.1093/jac/dkaa448

Recacha, E., Machuca, J., Díaz de Alba, P., Ramos-Güelfo, M., Docobo-Pérez, F., Rodriguez-Beltrán, J., et al. (2017). Quinolone Resistance Reversion by Targeting the SOS Response. *mBio* 8, 10.1128/mbio.00971-17. doi: 10.1128/mbio.00971-17

Thomason, L. C., Costantino, N., and Court, D. L. (2007). *E. coli* Genome Manipulation by P1 Transduction. *Current Protocols in Molecular Biology* 79, 1.17.1-1.17.8. doi: 10.1002/0471142727.mb0117s79
